# Pembrolizumab Plus Chemotherapy or Anlotinib *vs.* Pembrolizumab Alone in Patients With Previously Treated EGFR-Mutant NSCLC

**DOI:** 10.3389/fonc.2021.671228

**Published:** 2021-04-16

**Authors:** Ya Chen, Zhengyu Yang, Yanan Wang, Minjuan Hu, Bo Zhang, Yanwei Zhang, Fangfei Qian, Wei Zhang, Baohui Han

**Affiliations:** Department of Pulmonary, Shanghai Chest Hospital, Shanghai Jiao Tong University, Shanghai, China

**Keywords:** non-small cell lung cancer, pembrolizumab, epidermal growth factor receptor, antiangiogenic agent, chemotherapy

## Abstract

**Objectives:**

More and more encouraging evidence revealed that immunotherapy could improve clinical outcomes in patients with previously treated non-small cell lung cancer (NSCLC) with epidermal growth factor receptor (EGFR) variations. However, immunotherapy is still a controversy for NSCLC patients with EGFR mutation.

**Method:**

In this retrospective analysis, we compared the clinical efficacy of pembrolizumab monotherapy (PM), pembrolizumab combined with chemotherapy (P+C) and pembrolizumab combined with anlotinib (P+A) in NSCLC patients with EGFR mutation who had failed on EGFR-TKI and platinum-based chemotherapy.

**Result:**

Eighty-six patients were included in this study. The overall median progression free survival (PFS) was 3.24 months. Multivariate analysis suggested that EGFR*^L858R^* and combined therapy were positive prognostic factors of PFS. The overall median OS was 12.28 months. Multivariate analysis found that high PD-L1 expression (≥50%) and combined therapy seemed to be positive prognostic factors of OS. Among the population, 32 patients received PM, 26 patients received P+C and 28 patients received P+A. Up to Jan 30, 2021, the median progression-free survival was 1.5 months in the PM group, 4.30 months in the P+C group and 3.24 months in the P+A group. The median OS were 7.41, 14.92 and 15.97 months, respectively. The ORR were 3.1%, 23.1% and 21.4%.

**Conclusion:**

The addition of chemotherapy or antiangiogenic therapy to pembrolizumab resulted in significantly longer PFS, OS and ORR than pembrolizumab alone in our study. EGFR*^L858R^* might be a positive prognostic factor of PFS and high PD-L1 expression might be a positive prognostic factor of OS.

## Introduction

Targeted therapy has revolutionized the treatment landscape for patients with non-small cell lung cancer (NSCLC) with epidermal growth factor receptor (EGFR) mutations. Treatment with EGFR-TKIs, which have been developed to the third generation, provides better disease control and longer survival for patients with EGFR mutations (1). At the same time, screening for PD-L1 expression has become standard practice with the rise of immunotherapy. Of interest, the presence of EGFR mutations has been reported to upregulate the expression of PD-L1 (2–7). However, several studies have revealed that high PD-L1 expression predicted poor response to EGFR-TKIs in patients with EGFR-mutant NSCLC and correlated with primary resistance to EGFR-TKIs (8–10). Nevertheless, EGFR-TKIs have shown overwhelming advantages over standard chemotherapy in patients with EGFR-mutant NSCLC. EGFR-TKIs are recommended as the first-line treatment in this population according to the NCCN guidelines. However, treatment options after the development of TKI resistance need to be further explored. Given the growing emphasis on molecular profiling and detection of PD-L1, more detailed treatment guidance is needed for the critical population of patients with advanced NSCLC with both high PD-L1 and EGFR mutations.

Recently, the use of immune checkpoint inhibitors (ICIs) has greatly altered the standard of care for patients with advanced NSCLC without targetable EGFR or ALK genetic aberrations depending on the patient’s PD-L1 expression level. However, immunotherapy is still a controversial for patients with EGFR mutations because several clinical studies, including Checkmate057, Keynote010, POPLAR and OAK, have revealed that immunotherapy failed to improve clinical outcomes in patients with advanced NSCLC with EGFR mutations (11–15). However, the final OS data of the ATLANTIC trial showed that durvalumab improved clinical activity across all cohorts in patients with previously treated advanced NSCLC, including those with EGFR mutations (16). In addition, JAMA oncol reported that the combination of pembrolizumab plus docetaxel improved clinical outcomes in patients with previously treated NSCLC with EGFR variations (17). Furthermore, the ABCP group in the IMPOWER150 trial also prolonged the OS of patients with sensitive EGFR mutations (18). These encouraging results underline the necessity for further investigation into immunotherapy in patients with NSCLC with EGFR mutations. In this study, we collected the clinical records of patients with NSCLC with EGFR mutations who received pembrolizumab at our institution, including pembrolizumab monotherapy (PM), pembrolizumab combined with chemotherapy (P+C) and pembrolizumab combined with anlotinib (P+A). Anlotinib is an antiangiogenic agent that inhibiting VEGFR, FGFR, and PDGFR and has been approved by China National Medical Products Administration (NMPA) (19). In addition, it has been proved to be effective in NSCLC patients with EGFR mutations (20). In this study, we explored the efficacy of PD-1 in previously treated NSCLC patients with EGFR mutation.

## Material and Methods

### Patients

The medical records of patients with advanced NSCLC with EGFR mutations received pembrolizumab treatment at the Shanghai Chest Hospital between Dec 1, 2017 and Oct 30, 2020 were screened. Eighty-six patients met the following inclusion criteria: (1) stage IV NSCLC (2) positive EGFR mutation [exon 19 deletion mutation (EGFR*^D19^*), exon 21 L858R mutation (EGFR*^L858R^*), secondary exon 20 T790M mutation and other uncommon sensitive mutation such as G719X, L861R] (3) patients had disease progressed with at least 1 approved EGFR-TKI (patients with 20T790M mutation must had failed on osimertinib) and platinum-based chemotherapy following standard treatment guideline; (4) patients received PM, P+C or P+A (5) Eastern Cooperative Oncology Group performance status (ECOG PS) 0-1. Therapeutic schedule was decided by physician under the principle that patients at high risk of bleeding should not be treated with P+A, patients with severe adverse effects to previous chemotherapy should not chose P+C as priority. This study was approved by the Institutional Review Board of Shanghai Chest Hospital and performed following the declaration of Helsinki.

### Treatment and Clinical Response Evaluation

Among the three groups (PM, P+C and P+A), pembrolizumab was administered 200mg intravenously every 3 weeks. Chemotherapy was administrated following the standard NCCN guidelines. Chemotherapy regimens included docetaxel combined with carboplatin (DC) and nab-paclitaxel combined with carboplatin (TC). Antiangiogenic agent was anlotinib (given orally, 8mg once daily on days 1–14 of a 21-day cycle). Disease stage was decided on the 8th edition of the American Joint Committee on Cancer (AJCC) tumor-node-metastasis (TNM) classification. Enhanced chest computed tomography (CT) scan and abdominal ultrasound scan were examined every 4 weeks for therapeutic response evaluation. Enhanced brain magnetic resonance imaging (MRI) was examined every 4-6 months if no lesion at baseline and no symptoms thereafter. The response was evaluated according to the Response Evaluation Criteria in Solid Tumors (RECIST) version 1.1.

### Detection of Gene and Programmed Death Ligand 1 (PD-L1) Tumor Proportion Score (TPS)

The tissue sample was biopsied at the time of disease diagnosis and disease progression. EGFR detection was performed by the amplification refractory mutation system (ARMS) or by next generation sequencing (NGS). PD-L1 expression was assessed at the time of disease progression, right before the initiation of immunotherapy. TPS was detected by the PD-L1 IHC 22C3 pharmDx assay and was classified into TPS<0, 1-49% and ≥50%.

### Statistical Analysis

The χ2 test was used for comparison of categorical variables. The primary endpoints were PFS (from immunotherapy initiation to disease progression or the last follow-up); OS (from immunotherapy initiation to death or the last follow-up) and ORR (the ratio of complete and partial response). The median PFS and OS was estimated using the Kaplan-Meier method and compared by the log-rank test. Hazard ratios (HR) and 95% confidence intervals were estimated by a stratified Cox proportional-hazards model. To avoid the influence of confounding factors, factors with p values less than 0.1 in univariate analysis were included in multivariate analysis. All statistical analyses were performed using SPSS version 22.0 (IBM Corporation, Armonk, NY, USA).

## Results

### Clinical Features

Eighty-six patients who met the eligibility criteria were included in this study. Of these, most patients were male (55.8%) and non-smoker (52.3%) and had received third or more lines of therapy (**Table 1**). 17 patients (19.8%) had brain metastasis. The most common EGFR mutation type was EGFR*^L858R^* (54.6%), followed by EGFR*^D19^* (25.6%), uncommon sensitive mutation (10.5%) and T790M (9.3%). 75 patients were screened for PD-L1 expression levels immediately before immunotherapy, 17 (19.8%) of whom had a TPS of 0%, 32 (37.2%) of whom had a TPS of 1-49% and 26 (30.2%) of whom had a TPS of 50% or greater.

**Table 1:** Clinicopathological characteristics of 86 EGFR-mutated patients treated with pembrolizumab.

Characteristics	Number	Percent (%)
Age(median age, Range)	62(39-80)	–
Sex		
Male	48	55.8%
Female	38	44.2%
Smoking history		
Yes	41	47.7%
No	45	52.3%
Recurrence after surgery		
Yes	39	45.3%
No	47	54.7%
Treatment line of PD-1 Inhibitors		
Second line	4	4.7%
Third or after line	82	95.3%
Brain metastasis		
Yes	17	19.8%
No	69	80.2%
PD-L1 TPS		
<1%	17	19.8%
1~49%	32	37.2%
≥50%	26	30.2%
Unknown	11	12.8%
EGFR mutation subtype		
19del	22	25.6%
21L858R	47	54.6%
T790M	8	9.3%
Other	9	10.5%
Treatment		
PM	32	37.2%
P+C	26	30.2%
P+A	28	32.6%

### Progression−Free Survival

**Figure 1:**
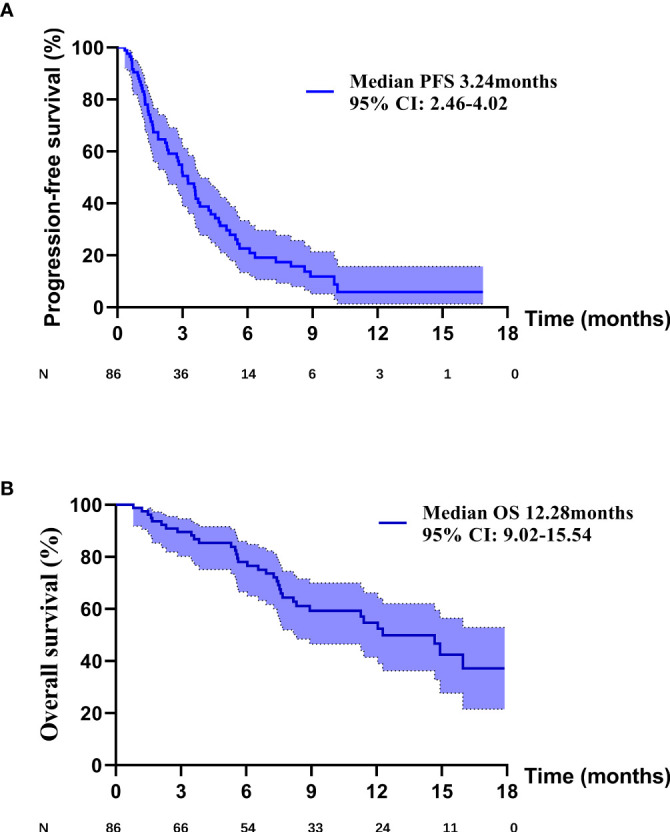
**(A)** The PFS curve of overall patients. **(B)** The OS curve of overall patients.

PD occurred in 64 (74.4%) patients in the overall population, including 26 (81.3%) patients in PM group, 12 (46.2%) patients in P+C group and 26 (92.9%) patients in P+A group. The overall median PFS was 3.24 months (95% CI: 2.46–4.02) (**Figure 1A**). Univariate analysis found that brain metastasis (p = 0.024), PD-L1 expression [p (1-49% vs 0) = 0.027, p (≥50% vs 0) =0.004)] and therapy [p (P+C vs PM) <0.001, p (P+A vs PM) = 0.002)] were associated with PFS (**Table 2**). Multivariate analysis found that patients with EGFR*^L858R^* had longer PFS than those with EGFR*^D19^* (p=0.024), patients in P+C group (p<0.001) and P+A group (p<0.001) had longer PFS than those in PM group (**Table 2**). These results suggested that EGFR mutation type and treatments were independent prognostic factors of PFS.

**Table 2:** Univariate and multivariable analyses for covariables associated with progression free survival.

Characteristics	Category	Univariate analysis HR (95 %CI)	p	Multivariate analysis HR (95 %CI)	p
Age	≤65 vs >65 years	0.71 (0.43-1.19)	0.194		
Sex	Male vs female	0.88 (0.53-1.46)	0.630		
Smoking history	Yes vs no	1.43 (0.88-2.35)	0.153		
Treatment line	Second line vs posterior line	1.15 (0.36-3.68)	0.814		
Brain	Yes vs No	1.40 (1.05-1.87)	0.024	0.74 (0.39-1.41)	0.361
PD-L1 expression	1-49% vs 0	0.46 (0.23-0.92)	0.027	0.47 (0.22-1.03)	0.058
	≥50% vs 0	0.33 (0.16-0.70)	0.004	0.46 (0.21-1.02)	0.057
	Unknown vs 0	0.71 (0.32-1.61)	0.414	0.60 (0.25-1.42)	0.246
EGFR mutation	21L858R vs 19del	0.60 (0.33-1.09)	0.092	0.41 (0.19-0.90)	0.024
	T790M vs 19del	0.83 (0.33-2.11)	0.691	0.47 (0.17-1.26)	0.133
	Others vs 19del	0.47 (0.19-1.14)	0.095	0.38 (0.14-1.04)	0.059
Therapy	I+C vs IM	0.22 (0.11-0.45)	<0.001	0.16 (0.07-0.37)	<0.001
	I+A vs IM	0.41 (0.23-0.73)	0.002	0.31 (0.16-0.57)	<0.001
	I+C vs I+A	0.53 (0.27-1.06)	0.071	0.55 (0.25-1.19)	0.126

### Overall Survival

Death occurred in 35 (40.7%) patients in the overall population, including 18 (56.3%) patients in PM group, 6 (23.1%) patients in P+C group and 11 (39.3%) patients in P+A group. The overall median OS was 12.28 months (95% CI: 9.02–15.54) (**Figure 1B**). Univariate analysis found that PD-L1 expression [p (≥50% vs 0) =0.007)] and therapy [p (P+C vs PM) =0.021, p (P+A vs PM) =0.020)] were associated with OS (**Table 3**). Multivariate analysis, including brain metastasis, PD-L1 expression and therapy, found that patients with high PD-L1 expression (≥50%) had longer OS than those with negative expression (p=0.039), patients in P+C group (p=0.035) and P+A group (p=0.019) had longer OS than those in PM group (**Table 3**). Hence, high PD-L1 expression and combined therapy seemed to be positive prognostic factors of OS.

**Table 3:** Univariate and multivariable analyses for covariables associated with overall survival.

Characteristics	Category	Univariate analysis HR (95 %CI)	p	Multivariate analysis HR (95 %CI)	p
Age	≤65 vs >65 years	0.65 (0.32-1.22)	0.168		
Sex	Male vs female	0.67 (0.33-1.35)	0.264		
Smoking history	Yes vs No	1.50 (0.76-2.96)	0.242		
Treatment line	Second line vs posterior line	0.47 (0.14-1.54)	0.211		
Brain	No vs Yes	0.51 (0.24-1.09)	0.082	1.31 (0.88-1.93)	0.180
PD-L1 expression	1-49% vs 0	0.64 (0.30-1.59)	0.339	0.94 (0.35-2.48)	0.895
	≥50% vs 0	0.22 (0.07-0.67)	0.007	0.30 (0.10-0.94)	0.039
	Unknown vs 0	1.74 (0.67-4.55)	0.257	2.27 (0.84-6.15)	0.107
EGFR mutation	21L858R vs 19del	0.79 (0.36-1.72)	0.557		
	T790M vs 19del	0.83 (0.32-4.32)	0.802		
	Others vs 19del	0.58 (0.18-1.86)	0.361		
Therapy	I+C vs IM	0.34 (0.13-0.85)	0.021	0.35 (0.13-0.93)	0.035
	I+A vs IM	0.41 (0.19-0.87)	0.020	0.40 (0.19-0.86)	0.019
	I+C vs I+A	0.82 (0.30-2.23)	0.700	0.88 (0.31-2.46)	0.807

### Survival Analysis of Patients in Different Therapy Group

**Figure 2:**
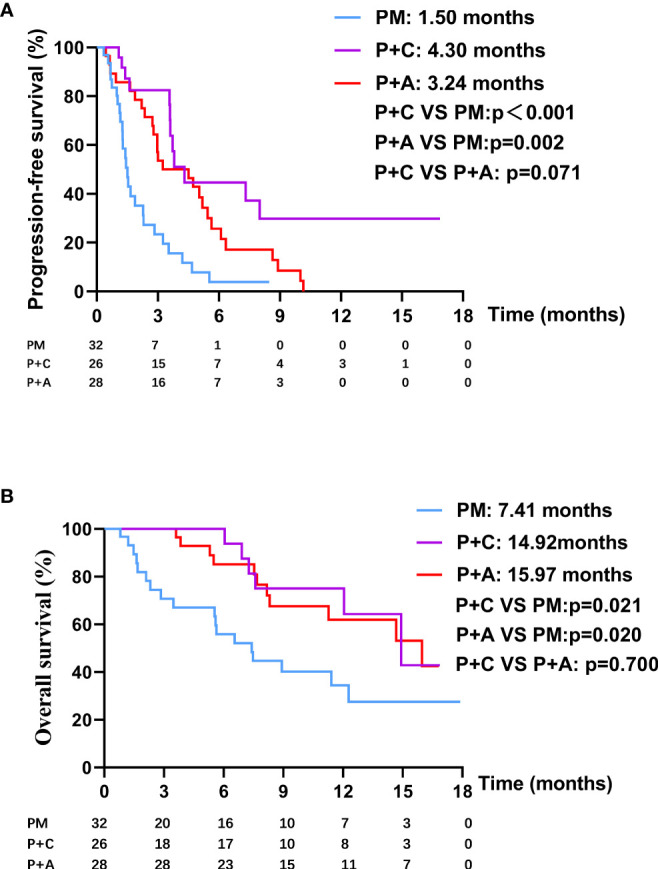
**(A)** The PFS curve of patients in PM, P+C and P+A groups. **(B)** The OS curve of patients in PM, P+C and P+A groups.

**Figure 3:**
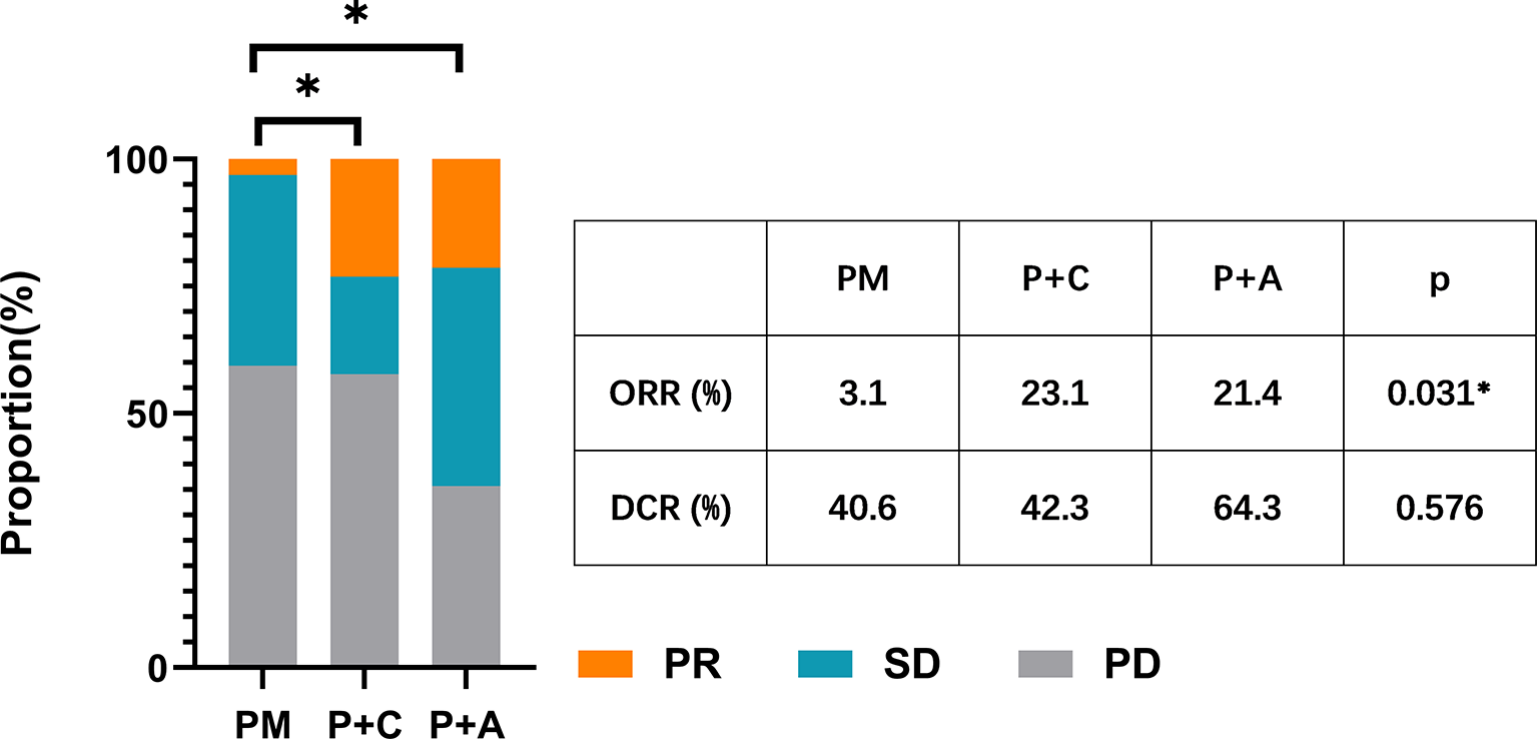
The ORR and DCR of patients in PM, P+C and P+A groups. * represents a statistically significant difference.

We divided the patients into three groups according to the therapy they received (32 patients in PM group, 26 patients in P+C group and 28 patients in P+A group). The baseline characteristics among the three groups were shown in **Table 4**. There were no statistically significant differences in characteristics among the three groups, indicating that no large selection bias existed. The median PFS was 1.5 months (95% CI: 1.19-1.81) in the PM group, 4.30 months (95% CI: 3.21-5.39) in the P+C group and 3.24 (95% CI: 0.96-5.52) months in the P+A group (**Figure 2A**). The median OS of PM, P+A and P+C were 7.41 (95% CI: 4.30-10.52), 14.92 (95% CI: 9.75-20.09) and 15.97 (9.57-22.37) months, respectively (**Figure 2B**). P+C group showed a significant PFS and OS benefit over PM group (p<0.001 and p=0.021). P+A group also revealed a significant PFS and OS benefit over PM group (p=0.002 and p=0.020). The ORR of PM, P+C and P+A group were 3.1%, 23.1% and 21.4% (**Figure 3**). The difference of objective tumor response showed us the superiority of combined therapy over monotherapy [P+C vs PM (p=0.038), P+A vs PM (p=0.041)]. The DCR were 40.6%, 42.3% and 64.3%.

**Table 4:** Clinicopathological characteristics of all patients with different treatments.

Characteristics	Monotherapy (N=17) (%)	With chemotherapy (N=15) (%)	With anlotinib (N=14) (%)	p value
Age				
Median, range	61(39-80)	66(54-78)	59 (41-78)	0.081
Sex				0.763
Male	19 (59.4)	13 (50.0)	16 (57.1)	
Female	13 (40.6)	13 (50.0)	12 (42.9)	
Smoking history				0.471
Yes	18 (56.2)	11 (42.3)	12 (42.9)	
No	14 (43.8)	15 (57.7)	16 (57.1)	
Recurrence after surgery				0.800
Yes	16 (50.0)	11 (42.3)	12 (42.9)	
No	16 (50.0)	15 (57.7)	16 (57.1)	
Treatment line of PD-1 Inhibitors				0.862
Second line	2 (6.3)	1 (3.8)	1 (3.6)	
Third/after line	30 (93.7)	25 (96.2)	27 (96.4)	
Brain metastasis				0.700
Yes	6 (18.8)	5 (19.2)	6 (21.4)	
No	26 (81.2)	21 (80.8)	22 (78.6)	
PD-L1 TPS				0.131
<1%	8 (25.0)	1 (3.8)	8 (28.6)	
1~49%	13 (40.6)	13 (50.0)	6 (21.4)	
≥50%	9 (28.1)	8 (30.8)	9 (32.1)	
Unknown	2 (6.3)	4 (15.4)	5 (17.9)	
EGFR mutation subtype				0.152
19del	5 (1.6)	6 (23.1)	11 (39.3)	
21L858R	21 (65.6)	17 (65.4)	9 (32.1)	
T790M	3 (9.4)	1 (3.8)	4 (14.3)	
Others	3 (9.4)	2 (7.7)	4 (14.3)	

### Subgroup Analysis of Patients in P+C and P+A Group

**Figure 4:**
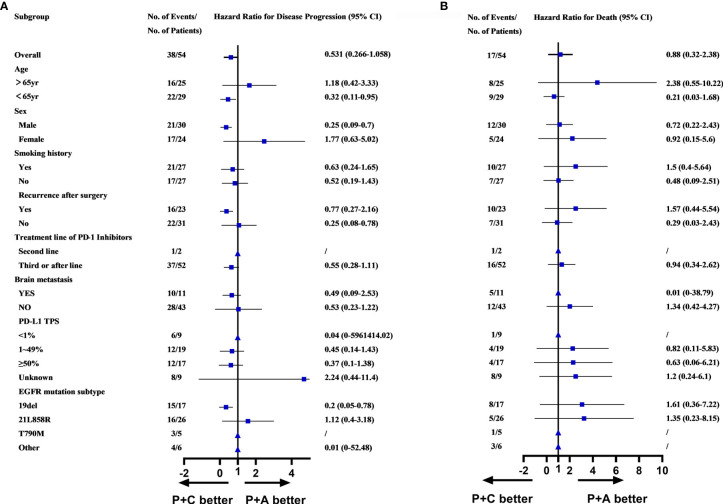
**(A)** Subgroups analysis of PFS in P+C and P+A groups. **(B)** Subgroups analysis of OS in P+C and P+A groups. (▲Represented HR cannot be calculated due to the sample.

Survival analysis, including PFS, OS and ORR had demonstrated the superiority of combination therapy (P+C and P+A) over monotherapy (PM). However, no significant difference was found between P+C and P+A. We conducted a subgroup analysis of the patients in the P+C and P+A groups to determine the specific characteristics of each treatment. The subgroup analysis of the PFS showed that patients of <65years old (HR=0.32, 95%CI: 0.11-0.95), male patients (HR=0.25, 95%CI: 0.09-0.70), patients that relapsed after surgery (HR=0.25, 95%CI: 0.08-0.78) and patients with EGFR*^D19^* (HR=0.20, 95%CI: 0.05-0.78) preferred P+C to P+A (**Figure 4A**). However, no difference was found in OS subgroup analysis (**Figure 4B**).

## Discussion

To our knowledge, this is the first study to directly compare the efficacy of PD-1 inhibitor monotherapy, PD-1 inhibitor plus chemotherapy and PD-1 inhibitor plus antiangiogenic agents in patients with previously treated advanced NSCLC with EGFR mutations. In this retrospective study, the addition of chemotherapy or anlotinib to pembrolizumab resulted in significantly prolonger PFS, OS and ORR compared with pembrolizumab alone. The median PFS and OS of the whole population in the present study were 3.24 and 12.28 months, respectively. Of interest, the univariate analysis and multivariate analysis found that combined therapies (P+C and P+A) were positive prognostic factors for both the PFS and OS.

The feasibility of immunotherapy for patients with EGFR mutation has long been controversial. A phase II study (NCT02879994) of pembrolizumab in TKI naive patients with EGFR mutations, advanced NSCLC and PD-L1-positive tumors was suspended due to lack of efficacy, which indicating that pembrolizumab was not suitable as a first-line treatment in this population (21). Besides, Checkmate057, Keynote010, POPLAR and OAK trials showed us the poor efficacy of PD-1/PD-L1 monotherapy in patients with EGFR mutations who had progressed after platinum-based doublet chemotherapy and treatment with EGFR-TKIs (12–15). However, the final overall survival update of the ATLANTIC trial demonstrated a promising OS benefit across all cohorts, especially in patients with EGFR mutations (16). The median OS of patients with NSCLC with EGFR mutations (TPS ≥ 25%) was 16.1 months, which was longer than that observed in patients with TPS ≥ 25% EGFR−/ALK− tumors (median OS of 10.9 months) (16). Of note, the median PFS and OS following PM treatment in our cohort were 1.50 and 7.41 months, respectively, which was consistent with the Keynote010 trial and failed to copy the success of ATLANTIC. This might be due to the difference between PD-1 inhibitors and PD-L1 inhibitors (12).

Recently, Christian et al. reported the results of a phase II clinical trial evaluating the effect of ICI (pembrolizumab) plus chemotherapy (docetaxel) vs chemotherapy (docetaxel) alone in previously treated patients with advanced NSCLC, including patients with sensitizing EGFR mutations who had experienced disease progression after platinum-based chemotherapy. For patients with EGFR variations, the PFS (6.8 vs 3.5 months) and ORR (58.3% vs 23.1%) were statistically significantly different in favor of the combination arm, which highlighted the efficacy of the combination of immunotherapy and chemotherapy. Similarly, another phase II study of immunotherapy (toripalimab) plus chemotherapy in patients with EGFR-mutant advanced NSCLC patients found that the combined treatment yielded encouraging PFS (7.0 months) and ORR (50%) (22). In our cohort, the median PFS and ORR of P+C treatment were 4.30 months and 23.1%, respectively. Our cohort’s PFS rate and ORR were lower than those reported in the aforementioned, which probably because our patients were more heavily treated. Nevertheless, our study verified the benefit of the combination treatment.

The IMPOWER150 trial revealed encouraging PFS and OS following immunotherapy, albeit in a combination therapy pattern, in patients with NSCLC with EGFR mutations (18, 23). The OS was greater in the ABCP arm (29.4 months) than in the BCP arm (18.1 months) but the difference was not statistically significant (HR, 0.60; 95% CI, 0.31-1.14), which might be due to the study’s small sample size, which might due to the small sample (24). In contrast, the IMPOWER130 trial revealed no significant PFS or OS benefit for patients with EGFR and ALK alterations. This difference in results highlighted the necessity of antiangiogenic agents and supported the hypothesis that antiangiogenic agents could enhance immune efficacy, which might be due to the remarkable improvement of antigen-specific T-cell migration, in patients with NSCLC with EGFR mutations in response to antiangiogenic treatment (25). Similarly, the combination of ICIs and antiangiogenic agents in our cohort also yielded greater PFS (HR, 0.41; 95% CI, 0.23-0.73) and OS (HR, 0.41; 95% CI, 0.19-0.87) than ICIs alone. Meanwhile, Zhai et al. found that anlotinib combined with PD-1 inhibitors showed promising efficacy as a third- or further-line treatment for NSCLC (26). The combination treatment achieved a median OS of 17.3 months, which was similar to the OS of 15.97 months in the P+A group in our cohort. This encouraging result suggested that the combination of immunotherapy and antiangiogenic agents might overcome the barriers associated with immunotherapy for patients with EGFR- mutant NSCLC patients.

Our study demonstrated the superiority of combination therapy (pembrolizumab plus anlotinib or pembrolizumab plus chemotherapy) intuitively. However, was found in the survival analysis between the group that received P+A and the group that received P+C. Future research studies with a larger sample size are needed to define the subgroups of patients to determine precise treatment strategies.

Hastings, K. et al. found that different EGFR mutation subtypes responded to ICIs differently (27). EGFR*^L858R^* resulted in longer PFS and OS than EGFR*^D19^*, which might be due to the higher tumor mutation burden (TMB) in the EGFR*^L858R^* group. Consistent with the previous findings, our multivariate analysis in our study also found that EGFR*^L858R^* was associated with a longer PFS than EGFR*^D19^* (HR:0.41, p=0.024). However, no OS benefit of EGFR*^L858R^* was found, which might be due to the small sample size.

There is no doubt that the expression level of PD-L1 is correlated with the efficacy of pembrolizumab in patients with NSCLC without EGFR mutations (28). Meanwhile, several studies have found that patients with EGFR mutations and PD-L1+ were more likely to respond to ICI monotherapy or ICI plus chemotherapy than those who were PD-L1 negative (22, 29, 30). However, some studies did not address the problem that chemotherapy and EGFR-TKIs might affect the expression of PD-L1. Hence, we utilized the tumor samples that were re-biopsied immediately before immunotherapy to detect PD-L1 expression to reduce bias (31, 32). We assessed the relationship between the efficacy of ICIs and PD-L1 expression and revealed that patients with PD-L1≥50% had longer OS than the OS of the PD-L1 negative group (HR:0.30, p=0.039).

Our study is limited by its retrospective nature. First, the data was collected from one center and the sample size was relatively small. Also, selection bias existed inevitable due to unavoidable missing data. However, the baseline clinical characteristics of patients in the PM, P+C and P+A group were balanced well, indicating that no large selection bias existed. Additionally, the heterogeneity of PD-L1 expression within tumors was inevitably existed though all detection were performed under guideline.

In summary, our analysis revealed that pembrolizumab plus chemotherapy or antiangiogenic agents could significantly prolong the PFS, OS and ORR compared with those observed following treatment with pembrolizumab alone in previously treated patients with advanced NSCLC with EGFR mutations. Our findings highlight the efficacy of the combination strategy of immunotherapy in this specific population. We also found that immunotherapy might be a more promising therapeutic agent for patients with EGFR*^L858R^* and patients with PD-L1≥50%. Based on the current findings, we hold the opinion that relevant clinical trials are urgently needed. The efficacy and safety of immunotherapy plus chemotherapy or antiangiogenic therapy in patients with EGFR-mutant NSCLC, especially those with high PD-L1 expression, should be further explored in clinical trials that provide strong evidence-based medicine data.

## Data Availability Statement

The datasets presented in this article are not readily available because of the following: ethical requirements for Shanghai chest hospital. Requests to access the datasets should be directed to 18930858216@163.com.

## Author Contributions

YC, ZY, and YW have substantial contributions to the conception or design of the work, the collection and analysis of data, and the writing and editing of the article. The rest authors have given substantial contributions to the work by providing editing and writing assistance. All authors contributed to the article and approved the submitted version.

## Funding

This research was supported by Shanghai Xuhui District municipal health commission [grant number XHLHGG201806] and Shanghai Shenkang three-year project[grant number SHDC 2020CR4017].

## Conflict of Interest

The authors declare that the research was conducted in the absence of any commercial or financial relationships that could be constructed as a potential conflict of interest.
